# Curating the world’s peer-reviewed literature

**DOI:** 10.1308/003588412X13171221591132

**Published:** 2012-05

**Authors:** David A Rew

**Affiliations:** Department of Surgery,University Hospital Southampton NHS Foundation TrustSouthampton, UR

**Keywords:** Research, Peer Review, Search Engine, Bibliometrics

## Abstract

New technologies are transforming academic publishing, including surgical research. The author considers a variety of the new systems, platforms and search engines that are fuelling this information revolution, as well as the bibliometrics and citation analysis necessary for filtering quality material for the increasingly inundated researcher.

The internet has spawned a new world of technologies and information systems that are transforming the international academic publishing environment. This provides new opportunities in professional communication for authors, re viewers, editors and publishers. There are also significant challenges, especially those related to managing the huge volume of available information and assessing its validity and quality.

We are all now familiar with the luxury and immediacy of web-based literature searches, using ‘free at the point of use’ systems such as Google, Google Scholar and PubMed; controlled access public search systems such as NHS Athens; and commercial/institutional services such as the Web of Science, provided by Thomson Reuters, and Scopus, provided by Elsevier.

The world’s published literature includes up to 500,000 periodicals of all types of academic and scholarly subject areas, as listed in the authoritative Ulrich™ Global Serials Directory, of which some 80,000 or more are believed to be active peer-reviewed publications. Precise calculations are difficult because journals rise, fade and fall in many places and many languages. The Thomson Reuters Web of Knowledge, from which Impact Factors are currently calculated, contains some 10,000 active journals, while the more recently compiled Elsevier Scopus system includes around 20,000 journals, of which some 5,000 are in the health and life sciences and related subject areas.

## The measurement of publication quality

Such is the deluge of scientific papers appearing on the web that the individual researcher would be inundated, without the resources to sort the quality material from the flotsam and jetsam. Just as editors and peer re viewers curate the quality of individual manuscripts, so there is a need to curate journals to defined quality thresholds so that readers and authors can have confidence in finding quality content in finite time. Quality has a number of dimensions, high among which are trustworthiness, originality and readability. Just as Google selects quality material through hierarchical page rank algorithms, so individual authors, papers and journals acquire the reputation for quality with time through usage and citation (referencing in other articles and journals). Citation is a complex subject and the science of bibliometrics has grown up to analyse and study it.

Commercial citation systems represent a massive financial investment in computer power, in programming skills, in bibliographic collation of journals and content from numerous publishers and in sophisticated bibliometric analysis of this content. These systems are economically viable because they offer a comprehensive range of specialist features to purchasers and users, including governments, universities, and public and private companies.

At the single user level, citation analysis systems allow efficient literature searches on subjects, authors and keywords, and direct access in many instances to the full text of published content. Increasingly, they allow seamless navigation through reference lists to relevant cited papers and they allow a range of metrics to be obtained on authors and papers.

At the corporate level, the citation systems contain so much information that they are used to inform the ‘gross intellectual product’ of the university or country; to identify strengths and weaknesses in different disciplines; and to recruit productive researchers. So powerful is this process that Scopus has been selected to inform the 2015–2014 UR Research Excellence Framework (previously known as the Research Assessment Exercise). An understanding of how this system works may be helpful to academic surgeons whose research output will be assessed in these ways.

The UR National Research and Assessment Exercise ran in 1986, 1992, 1996, 2001 and 2008, as an increasingly sophisticated and demanding tool for the assessment of quality and value for money of academic investment and activity across UR higher education institutions and disciplines. A pilot study in advance of the forthcoming round concluded that ‘For science, engineering, technology and medicine, a combination of research income, postgraduate research student data and a bibliometric indicator of quality will be used to assess research’. Web of Science and Scopus were tested as bibliometric data resources. Scopus was found to have broader citation coverage by a range of measures and categories, including early career researchers, and was selected to inform the 2015–2015 Research Excellence Framework.

## The work of the Scopus Content Selection Advisory Board

Recognition and affiliation to the major citation indices has become an important hallmark of quality for peer reviewed journals. In 2009 Elsevier appointed an international Content Selection and Advisory Board of experienced journal editors from the sciences, arts and humanities as subject chairs, to brainstorm a wide range of quality issues in academic publication.^1^^,^^2^ We have developed the Scopus Title Evaluation Platform (STEP), which allows us to process online the many hundreds of applicant journals each year against objective criteria. It also rapidly became apparent that the Scopus STEP process could be a powerful tool for improvement of the world’s literature, using quality measures to help raise standards in publication worldwide in a number of ways.^3^

The first example of raising standards is a mandated statement of conformity on publication ethics and malpractice in every applicant and resident journal in Scopus. This will ensure that the excuse of ignorance is no longer tenable for any author, editor or publisher in any jurisdiction from now on.

The second example relates to language. An international citation system that simply cited material in the native language of the authors and editors would create a series of several hundred linguistic silos. A common and universal language can bring the most obscure paper to the attention of a specialist audience worldwide and transform the citation metrics of the paper and of its authors. English is now well established as the world’s common second language of professional communication. We have therefore taken active steps to encourage journals which publish in other languages to co-publish their content in English on the web, as an aid to better communication and exposure of their content to international audiences, and hence as an aid to improving their competitive quality, content and bibliometrics.

For the general medical user and literature searcher, there is now a range of choice of of quality curated literature search, citation and bibliometric systems, each of which is in a state of continuous development and each of which is backed by substantial public or commercial financial resources and the intellectual firepower of software engineers and bibliometrics experts. PubMed is a convenient, public access citation, abstract and reference resource with US government funding. It draws on the resources of Medline, which is the US National Library of Medicine’s database of some 5,400 journals and run by the US National Centre for Biotechnology Information. Scopus and Web of Science are multidisciplinary search platforms with similar scale in the biomedical field but evolving functionality, quality filters, linkage and access to full text content. They are usually accessed through institutional (eg university library) subscriptions.

Academic surgery and surgeons now compete for attention in a transparent international marketplace for information and ideas. A better understanding of the workings of this digital melee will help position and advance the influence and reputation of UR surgery worldwide.
